# Spatially disaggregated population estimates in the absence of national population and housing census data

**DOI:** 10.1073/pnas.1715305115

**Published:** 2018-03-19

**Authors:** N. A. Wardrop, W. C. Jochem, T. J. Bird, H. R. Chamberlain, D. Clarke, D. Kerr, L. Bengtsson, S. Juran, V. Seaman, A. J. Tatem

**Affiliations:** ^a^WorldPop, Department of Geography and Environment, University of Southampton, Southampton SO17 1BJ, United Kingdom;; ^b^Flowminder Foundation, SE 11355 Stockholm, Sweden;; ^c^Population and Development Branch, United Nations Population Fund, New York, NY 10158;; ^d^Bill and Melinda Gates Foundation, Seattle, WA 98109

**Keywords:** population, census, remote sensing, geostatistics, surveys

## Abstract

Population numbers at local levels are fundamental data for many applications, including the delivery and planning of services, election preparation, and response to disasters. In resource-poor settings, recent and reliable demographic data at subnational scales can often be lacking. National population and housing census data can be outdated, inaccurate, or missing key groups or areas, while registry data are generally lacking or incomplete. Moreover, at local scales accurate boundary data are often limited, and high rates of migration and urban growth make existing data quickly outdated. Here we review past and ongoing work aimed at producing spatially disaggregated local-scale population estimates, and discuss how new technologies are now enabling robust and cost-effective solutions. Recent advances in the availability of detailed satellite imagery, geopositioning tools for field surveys, statistical methods, and computational power are enabling the development and application of approaches that can estimate population distributions at fine spatial scales across entire countries in the absence of census data. We outline the potential of such approaches as well as their limitations, emphasizing the political and operational hurdles for acceptance and sustainable implementation of new approaches, and the continued importance of traditional sources of national statistical data.

Accurate population data at local levels are fundamental for a broad range of applications by governments, nongovernmental organizations, and companies, including the planning and delivery of services, election preparation, estimation of populations at risk for infectious disease or hazards, and disaster relief operations (1–6). The main sources for such demographic data are the national population and housing census, typically conducted once every 10 y (7), as well as national registers of births and deaths (8). However, in resource-poor settings, national registers are generally lacking or incomplete (9, 10). In many countries the reliability of population and housing census data has been questioned due to the accuracy of projections required from long delays between enumeration and data release (Fig. 1), the omission or undercounting of certain marginalized groups (e.g., those in informal settlements, ethnic minorities, and nomadic populations), insecurity and conflict limiting enumeration in certain regions, and corruption driving inflated estimates where population numbers are linked to resource allocation.

**Fig. 1.**
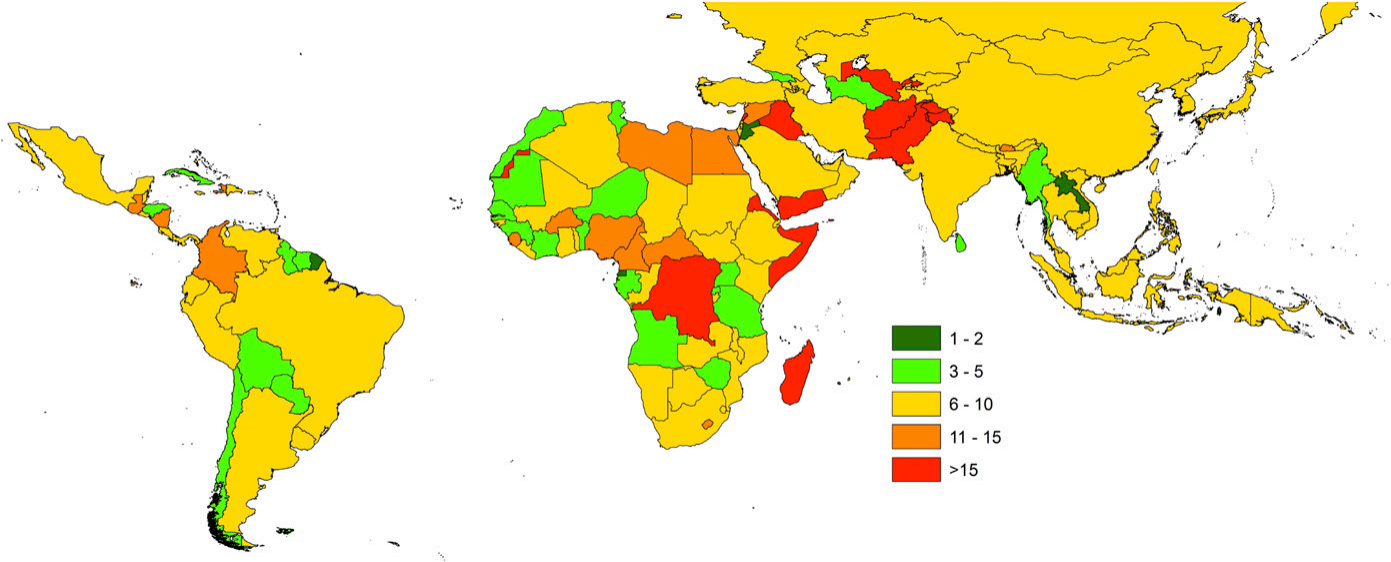
At the beginning of 2017, the number of years since the last national census in countries across Latin America, Africa, and Asia. Data from https://unstats.un.org/unsd/demographic/sources/census/censusdates.htm.

At local scales, further challenges limit the utility and accuracy of available population data. Census data, when linked with accurate, small-area administrative boundary data, can provide spatially explicit evidence based on population (10), but for privacy reasons available data that can be linked to the smallest administrative areas are limited. The availability and accuracy of the administrative boundaries available in digital formats and at fine spatial scales can also be variable, leading to a potential misallocation of populations spatially. Moreover, human populations are not uniformly distributed within areal units and thus aggregate population data, particularly when only available for larger areas, do not accurately represent the true spatial distribution of the population (4, 11). Consequently, accurate population data are often lacking in places where they are needed the most by governments and other organizations. However, recent advances in computing power, availability of regularly updated high-resolution satellite imagery, global positioning systems (GPS)-enabled field survey techniques, and statistical methods are presenting opportunities for alternative approaches to producing reliable, spatially refined estimates of human populations.

## The Need for High Spatial-Resolution Population Data

The lack of recent, reliable, and spatially detailed population data were highlighted with the 2013–2016 Ebola outbreak in West Africa, where emergency responders struggled to identify the location and size of rural settlements, and could not accurately calculate infection rates since the denominator (i.e., the population at risk) was not known, an issue that is regularly encountered in emergencies and outbreak situations (1, 12–14). Nearly all public health outreach efforts, from vaccinations to bed nets to HIV treatment, depend on accurate target population denominators to estimate resource needs and project costs, as well as to measure and assess results and impacts. International development goals are based on ensuring that a certain percentage of the population has access to specific services or resources, or achieves a certain level of social, economic, or physical health. These measurements require a solid and regularly updated understanding of not only how many people live in a country, but where and who they are (15).

As the move has been made from the Millennium Development Goals to the Sustainable Development Goals, there is now an explicit focus to leave no-one behind by reducing inequalities both between countries and within countries (15). This highlights the important aspect of within-country heterogeneity, whereby aggregated data may hide significant subnational disparities. However, assessing progress against Sustainable Development Goal indicators relies on the availability of standardized and robust data, including a reliable baseline population estimate from which to measure change (16). Given the importance of regional heterogeneity in population characteristics, the United Nations has explicitly called for improved availability of high-quality, timely, and reliable data disaggregated by income, gender, age, race, ethnicity, migratory status, disability, geographic location, and other characteristics relevant to national contexts. This will be vital to ensure subnational variation in indicators is adequately captured (17). Previous work to produce subnational population data has focused on approaches to disaggregate population counts from census-defined areal units to high spatial-resolution grids.

## Disaggregation of Administrative Unit-Based Population Data

**Fig. 2.**
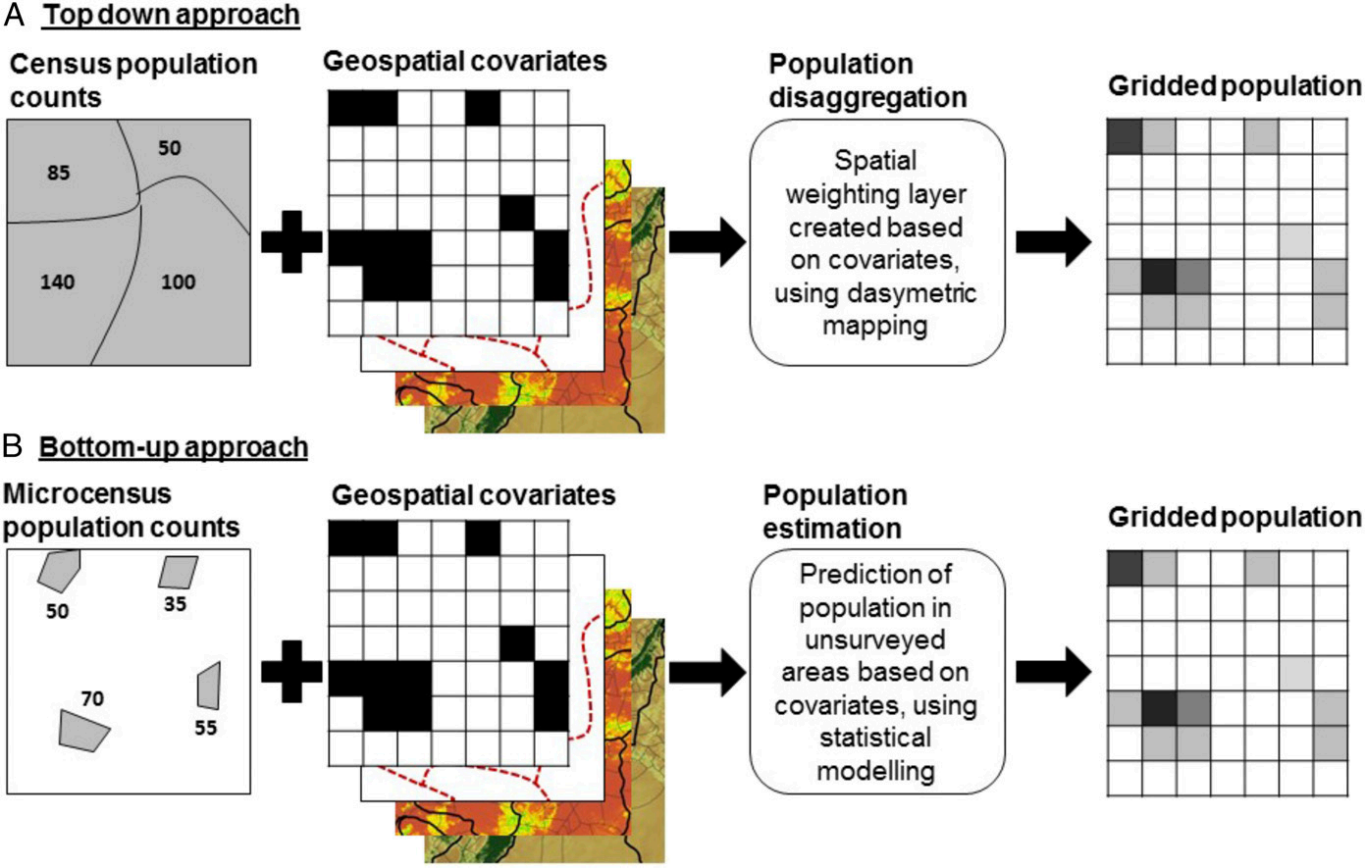
Schematic of (*A*) top-down and (*B*) bottom-up mapping approaches. Populations are assumed to be restricted to areas containing residential buildings; population density within these areas is predicted based on ancillary datasets such as road networks, temperature, or green vegetation.

Census data are typically made publically available aggregated in space by large administrative areas, typically districts or subdistricts, and these areal units present analytical challenges for population studies. The boundaries of these units are often arbitrary for the demographic variables of interest. Census data are collected by enumeration area, typically designed to cover around 500 people and be small enough for enumerators to cover in a day. Although enumeration area design generally aims to avoid significant differences in types of housing and population within units, they typically follow existing administrative boundaries and are designed for convenience of enumeration rather than to follow differences in population distribution. In an effort to characterize the spatial variation in the distribution of human populations and overcome the limitations of such aggregate data, much research has focused on creating alternative representations of population as a continuous surface (18). Dasymetric mapping techniques are well-known cartographic approaches [see Eicher and Brewer (19) and Mennis (18, 20)] to disaggregate areal unit representations to more spatially refined distributions. These techniques draw on ancillary or “covariate” data to redistribute data at finer scales by defining a functional relationship between, in this case, population density and the mapped ancillary data (21). This approach to population disaggregation, which we term “top-down” population mapping, is shown graphically in Fig. 2*A*. Since the mid-1990s (22) researchers have taken advantage of Geographic Information Systems and satellite remote-sensing technologies for dasymetric mapping of population, producing high spatial-resolution grids to represent ancillary data as well as for the resulting population distributions. These efforts have ranged from a simple equal weighting of census counts to grid cells within an administrative area, to the integration with higher spatial resolution covariate datasets and advanced statistical procedures, more accurately representing the distribution of the human population across space (4, 5, 11, 22, 23).

Early examples of top-down census disaggregation products include the proportional allocation of population counts to grid cells within each administrative area [Gridded Population of the World (GPW) V1] and smoothing across boundaries (GPW V1b) (22, 24), or allocation of population counts based on covariates known to correlate with population density, such as distance to major roads and land cover (e.g., LandScan 1998) (22, 23, 25). Over the past two decades, there have been considerable advances in data availability, computational power, and methodological approaches, enabling the production of gridded population datasets at finer spatial resolutions and global coverage (4, 11). Other advances include the use of very high-resolution satellite imagery and machine-learning techniques to identify human-built structures, with subsequent allocation of human population counts to these potentially settled sites (26). Further progress in this area will enable ever more accurate representations of human population distributions across space, at improved spatial resolutions, based on the availability of areal unit-based census and population estimate data.

## Challenges to “Top-Down” Disaggregation

Where high-quality, recent census data are available and linked with accurate digital boundary data that match with those used for enumeration, the disaggregation approaches described above can provide detailed and valuable representations of the spatial distribution of human populations. Prior research on population distribution models has typically focused on the challenge of selecting ancillary spatial data; however, the central challenge to the accuracy of top-down disaggregation methods is the reliability of input population data. Conducting a national census is an arduous, resource-intensive undertaking, and is a challenge even in countries with the necessary technology, infrastructure, and financial and human capacity (27). In low-income nations, or those that have undergone internal strife and frequent changes in government, maintaining accurate, up-to-date census data—particularly where population growth is rapid and nonuniform and migration rates are high—is an extraordinary challenge. It is therefore important to emphasize that gridded population estimates based on top-down disaggregation are only as good as the census data on which they are based.

A national census, which typically aims for complete enumeration of a population within a defined region or territory on a single specific date, can be challenging for a number of reasons. Capacity gaps in many national statistical offices, together with financial constraints, present challenges in many low income countries. In some cases, there may be a lack of political will or concerns that accurate and up-to-date population data may instigate instability. For example, Lebanon’s last population census was conducted in 1932 and there has been no recent census due to a complex combination of religious and sociodemographic concerns (28). Ongoing civil unrest, political instability, and war can prevent the planning and implementation of a national census. Enumeration may also be constrained in specific areas due to insecurity and lack of access to those regions. In these situations, estimated population growth rates provide the means for updating population data over time. The most recent census in Somalia, for example, was conducted between 1985 and 1986 and no data were publicly released. Thus, the only publically available census data for Somalia date from 1975 (29). In Afghanistan, the first and only national census was held in 1979, although this only covered 67% of districts due to insecurity (30). Further census enumeration was planned for 2008, with a precensus household listing exercise conducted between 2003 and 2005 (31). However, the census was rescheduled to 2011 due to insecurity, but was eventually cancelled because of the security situation, and has ultimately been replaced by a form of “rolling” sociodemographic and economic surveys, conducted in one or more provinces at a time (30). In Madagascar, the most recent census was conducted in 2003, with subsequent plans being postponed during a period of political instability (32), and a similar situation exists in the Democratic Republic of Congo, with new censuses since the last one in 1984 having often been postponed. Thus, while a total population census can provide the foundation to inform spatially disaggregated population estimates, it is clear that such estimates based on censuses that are decades-old, incomplete, or potentially biased by methodological or political processes need to be complemented by additional data sources.

## “Bottom-Up” Population Estimation

The growing requirement for spatially disaggregated population data and the absence of national population and housing census data in some countries mean that other data sources are increasingly being explored in efforts to produce spatially disaggregated population estimates at different geographical scales and time periods. We introduce here the concept of a bottom-up approach to population estimation, which shares a common goal with top-down approaches: to produce population estimates for small areas or uniform, high spatial-resolution grids. By aggregating these high-resolution predictions, population totals can also be produced for administrative units or for the national level if required. Conceptually, bottom-up estimation of population relies on complete counts of population within small, defined areas (which we call “microcensus surveys”), selected across an area of interest, and collected relatively rapidly and at a fraction of the cost of a full national census. Statistical models are then used to link microcensus data to spatial covariate data, with full coverage over the regions of interest to predict population numbers in unsampled locations (Fig. 2*B*).

Early examples of bottom-up style, spatially disaggregated population estimation approaches focused mainly on urban settings, with the area of cities being used to estimate population sizes. For example, a global linear regression was used to quantify the relationship between the log of population count and the log of urban area (based on urban extents determined using night-time lights), followed by estimation of national populations based on the percentage of the population in each country thought to reside in urban areas (33). This general approach has been further developed via the incorporation of additional ancillary variables to improve the predictive performance, such as distance to roads, slope, age of community (27), satellite-derived variables (e.g., mean reflectance in specific bands) (34), vegetation indices, texture, and surface temperature (35). Thus, bottom-up methods can also learn and benefit from advances in geospatial data used in the top-down models discussed previously.

The majority of related research has focused on high-income settings, where the availability of high-quality spatial datasets is generally good. There has been considerably less attention paid to low- and middle-income settings, where alternative population estimation approaches could provide a means to fill significant data gaps. However, access to high-quality spatial datasets (e.g., building function, footprint, or floor space data) in these areas tends to be more limited. Where similar methods have been applied in low- and middle-income settings, they have demonstrated strong potential. For example, satellite images of Quito were classified by density of buildings and microcensus surveys were undertaken to assess the feasibility and costs of population estimation (36). In an urban setting in Sierra Leone, population sizes were predicted using the number of buildings or rooftop areas (based on a combination of ground-based mapping and satellite imagery interpretation) (12, 37). Similarly, previous work in Minas Gerais, Brazil sought to estimate urban populations based on habitable surface areas (building footprints combined with building height data from laser scanning) (38). Population estimations for internally displaced persons or refugee camps have also been conducted in several countries, by simply multiplying the number of residential structures (based on manual interpretation of satellite imagery) with the average occupancy values; this approach resulted in good predictive accuracy in some, but not all, settings (39).

## Bottom-Up Population Estimation Methodologies

As illustrated in Fig. 2, the general bottom-up population estimation approach entails: (*i*) microcensus population enumeration; (*ii*) linkage with relevant ancillary datasets; and (*iii*) definition of the functional relationships between ancillary datasets and the population data to predict populations in unsurveyed locations.

### Microcensus Surveys.

Population data for a sample of areas across the area or country of interest are needed as a primary input to bottom-up population estimation. These data may come from a partial census, census-like population survey (i.e., where a survey is designed to provide population counts), or a specifically designed microcensus survey. The design of the microcensus (or other) survey used to provide training data is a vital step to ensure accurate and unbiased population predictions. The survey design should capture as best as possible the range of densities, demographics, and environments that exist across the area of interest, providing a representative sample. Consideration (for example using stratified sampling) should be given to geographical, socioeconomic, or environmental factors that may influence population densities within the specific context in which the work is being conducted. For example, urban areas are more densely populated than rural; average household sizes may vary according to ethnic, religious, or cultural groups; and the ruggedness of terrain may also influence settlement patterns and population density. The importance of these and other factors will vary by setting, so survey design should be conducted carefully, building on a detailed understanding of the specific context. Another key requirement for these data are robust georeferencing of the geographical areas where population data has been acquired.

Population enumeration can be conducted within administrative units (e.g., census tracts) or within other arbitrarily designed polygons, as long as the population data are explicitly linked to the correct geographical area. Printed maps can be used as guides to identify the correct geographical areas, based on physical landscape characteristics (e.g., roads, mountains, rivers, buildings). However, technological advances now offer a range of more sophisticated methods for the accurate geographical positioning of enumeration activities. GPS can be used to ensure enumeration is occurring in the correct location, and the inclusion of GPS technology in smart phones and tablet computers can integrate navigation and the recording of geographical coordinates into enumeration activities, minimizing locational error and human effort.

### Covariates.

The covariates used for bottom-up population estimation should be (*i*) strongly correlated to population density and (*ii*) available consistently across all areas where the population estimation is required. Population estimation approaches have previously been categorized as utilizing the relationships between population and covariate data representing the following: (*i*) built-up areas, (*ii*) areas of specific land use types, (*iii*) counts of dwelling units, (*iv*) satellite-derived measures such as spectral radiance, or (*v*) socioeconomic or physical characteristics (34, 35, 40). In practice, integration of multiple of these elements is likely to result in the best predictive performance, as they each capture different facets relating to how population numbers and densities vary spatially. Although access to high-quality, spatially comprehensive datasets representing some of these characteristics has traditionally been difficult in resource-poor settings, advances in image-processing techniques, computational power, and the increasing availability of very high-resolution satellite imagery (of the order of <10-m spatial resolution) means that the production of high-quality covariates for many settings is increasingly feasible (41, 42). The mapping of human settlements and even individual buildings from a new generation of satellite imagery and aerial photography is providing detailed geospatial data on human settlement patterns, a key input (as settlement areas or dwelling counts within specified areas) for bottom-up population estimation (12, 43). Furthermore, semantic detail can also be used, such as the density of dwellings or inhabitants (35, 37), types of buildings (e.g., residential/nonresidential) (44), or types of settlement patterns (e.g., informal/formal), which can be distinguished using computer-vision and machine-learning approaches (43).

Other ancillary spatial datasets capturing features related to how humans are distributed on the landscape are already widely used to improve the accuracy of dasymetric mapping. A comparison of top-down population density models in 32 low- and middle-income countries found that covariates related to defining settlements, climate, topography, and ecology consistently explained the most variation in population density (45). Datasets representing factors, such as distance to roads, elevation, slope, and night-time lights (4) can also be integrated for the improvement of predictive accuracy for bottom-up population mapping (35). There has been substantial growth in the availability of volunteered geographic information (VGI), such as through OpenStreetMap, which can help address these issues by providing data such as settlement extents, building footprints, or the locations of roads or facilities (46, 47), and VGI has been utilized in dasymetric mapping previously (11, 48, 49). However, VGI data can be prone to spatial bias in completeness, with a tendency toward better data availability in urban areas and wealthier countries so should be used with care (50). Finally, novel data sources, such as those derived from mobile communications and social media, show potential for not only providing additional spatial covariates to improve the accuracy of both top-down and bottom-up modeling approaches (51), but also as a way to capture the daily, seasonal, and annual dynamics of populations (52–54).

### Statistical Approaches.

The goal of bottom-up models is to predict populations across large areas where data exist for only a small subset of the area. Less emphasis is placed on explaining the processes producing population distributions, as might be done in models projecting fertility, mortality, and migration rates. It is important to distinguish this goal because it has implications for understanding the methodological approach. First the associations with the covariate datasets are not interpreted causally, which might lead to simplistic environmental determinism arguments. The initial covariate selection, as discussed above, does identify covariates that are expected to be correlated with population density. While some ancillary data could be associated with population density or growth rates, the relationships are also seen as indirect markers of the variation in population, which reflects how human settlements modify and are constrained by the environment. Second, the statistical methods used in bottom-up models must be capable of drawing together multiple sources of data to build the model. These multiple data sources provide information not only from the data space of relationships between population and covariates, but also from their positions in space. Specifically, observations taken closer together in space tend to be more similar than those further apart. This feature, known as spatial autocorrelation, is common to our first-hand experiences of the world—populations cluster together in similar groups—but it violates assumptions of independence that underlie classic statistical methods. Explicit consideration of spatial structure, on the other hand, can substantially improve predictive outputs (55, 56) because spatially structured variation between microcensus areas that is not fully explained by the covariates is still a source of useful information for predictions. Future research in this area should aim to explicitly address spatial autocorrelation, which should provide improved predictive accuracy. Several options are available to ensure that residual spatial autocorrelation is appropriately dealt with. For example, spatially autoregressive models may be used for the predictive modeling of areal data, with a spatial adjacency matrix being used to ensure that the probability of values estimated is conditional on the level of values in neighboring areal units (57). Alternatively, where population data can be represented as spatial points (e.g., using the centroid of small areal units with linked population data), geostatistical methods that model residual autocorrelation as a function of distance between points may be applied (58).

There are few examples in the literature of areal-based spatially explicit models where prediction into unsurveyed locations is the main aim, but none, as far as we are aware, that have applied these methods to population estimation. Nevertheless, currently available software does include this functionality (59), and where applied, the inclusion of spatial dependency has resulted in improved predictive performance (60). Predicted populations from these types of areal-based methods would be explicitly associated with areal units, and the subsequent spatial disaggregation of predicted population counts (along with associated confidence intervals or SEs) can then be used to provide high spatial-resolution population estimates and an indication of predictive uncertainty. While there are no specific examples in the literature of these methods being applied for population estimation, the extrapolation of household survey data by these means has been used to provide bottom-up spatial predictions of population age structures (61). These geostatistical methods have utilized data from specific field surveys and household survey data (e.g., Demographic and Health Survey data), where spatial coordinates refer to the location where the survey was conducted (e.g., a point within a village, or a health center location), or the (normally spatially displaced) survey cluster centroid for national household survey data. Application for the prediction of population size or density would require careful consideration of how population data are represented most accurately as points: explicit geographically linked enumeration (i.e., using GPS supported enumeration hardware to geolocate all households enumerated) provides the ideal basis for this, enabling enumeration within small, uniformly sized, and well-defined areas. Predictive outputs from geostatistical methods could be provided on a grid at the same spatial resolution for which covariate datasets are available (or at a coarser spatial resolution if required), thus removing the need for subsequent spatial disaggregation.

The previous examples of bottom-up population estimation discussed have focused on the application of linear regression models of raw population counts, population densities, or the natural log of one of these values. For the modeling of population counts, the application of Poisson, negative binomial, or quasi-Poisson regression may be more appropriate, given that population counts are inherently positive integers. Note also that the spatial modeling approaches we discuss here are more commonly discussed in ecological studies to predict the abundance of plant and animal species (62) than in the demography or population studies literature.

### Validation of Outputs.

Because the primary objective of bottom-up estimation is prediction in nonsampled areas, validation of the spatially disaggregated population estimates is rare. Where results have been reported previously they indicate a good correlation between predicted and observed total population or population density values, particularly when considering larger administrative units. For example, an *R*^2^ value of 0.72 (squared correlation coefficient for observed vs. predicted counts, using independent testing data) was obtained by Harvey et al. (34), highlighting good predictive performance. Similarly, population predictions from a linear regression model developed using data from 10% of available census units in the Netherlands, along with building floor space or volume data, produced predictive errors (calculated as median absolute percentage error, based on the remaining 90% of available census units) of 18.3% at the smallest administrative level, 9.3% at the largest administrative level, and 0.5% at the national level (44). Several previous studies have identified persistent overestimation of population density in rural areas or areas where buildings are largely nonresidential (e.g., industrial sites) and underestimation of population density in high-density urban settings, particularly where multistory buildings are common (34, 35, 44). This suggests that contextual information, such as residential vs. nonresidential buildings or building height information, may improve predictions. The utility of such additional information has been highlighted in the Netherlands, where building footprint areas, building floor space areas (which incorporates multiple floors per building), and building volume (which incorporates building heights) were used as covariates in a predictive linear regression approach, with building floor space found to produce the most accurate predictions (44). However, not all studies assessed predictive performance against an independent testing dataset, and in the majority of examples, values were not back-transformed before accuracy assessment (i.e., validation was based on population density or the log of population density values rather than population counts) (27, 35). Both of these scenarios result in an overestimation of predictive accuracy and differences in accuracy assessment protocols make meaningful comparison of different modeling approaches impossible.

Future research should incorporate the comparison and validation of different methodologies to provide a clear understanding of predictive accuracy. While limited applications to date have indicated good predictive performance, robust testing and validation should be conducted in a range of settings. In particular, validation studies performed in countries with comprehensive and reliable census data coverage should be a priority. Population estimates can also be compared with other administrative data, such as vaccination records (61) or local population projections. This type of testing and validation is required to strengthen the evidence that bottom-up approaches can be considered as an appropriate means of generating robust population estimates in areas where complete census coverage is not possible.

## Limitations

The bottom-up approach described here has the potential to produce spatially disaggregated population estimates in situations without recent and reliable census data; however, the approach should be seen to complement a census or other enumeration work. We emphasize the complementary nature of the bottom-up approach because a full-enumeration census can include additional information on socioeconomic and demographic characteristics in the population. Several limitations to the approach need to be considered. These caveats are broadly related to: (*i*) data collection and analysis methods, and (*ii*) interpretation/use of the resulting estimates. The methods described here are based on microcensus data collections, and these microcensus data must be collected with the same care and rigor as a full census. The field protocol should, at a minimum, include multiple layers of supervision and quality control. External observers and postenumeration surveys confirming data collection should be used when possible, as in full enumeration censuses. All data should be checked for consistency and to assess potential underenumeration. The smaller number of enumerators needed for a microcensus (compared with a full national census) is an opportunity to improve oversight and training to prevent such data-collection errors. The increasing use of mobile devices (e.g., smartphones and tablets) with inbuilt georeferencing capabilities also provides opportunities for improved data quality and validation, by providing the means to collect enumeration data with associated date, time, and location information.

A second area of concern is related to the use and broader impacts of population-mapping activities. As noted above, making claims about a population total even using a national census is a highly political and contentious issue. The results affect all per capita rate estimates, shift political representation, and change claims to power or resources. While the substantial task of a full national census can only be undertaken by a central government, the bottom-up approach is potentially available to more analysts. On the one hand, this means that typically marginalized or undercounted communities could produce alternative population estimates themselves; it also means that people from outside a country can make competing claims about a population. For this reason, we recommend openness and transparency in data sources and methods and for communication among analysts conducting a bottom-up mapping project and multiple stakeholders, including the central government.

Basing a country’s population estimate on a bottom-up statistical model invites questions about the accuracy of the model, particularly in situations without a full census for comparison. Country settings that lack an updated census may be experiencing conflicts, environmental hazards, or large population displacements. Such events make it difficult to accurately collect information on the highly mobile population, and misrepresenting populations at risk should be avoided. Uncertainty in the estimates is not inherently a limitation, however. Despite being portrayed as definitive and authoritative, a full census may contain errors. An advantage of the bottom-up estimation approaches are that uncertainty about population estimates can be explicitly quantified. The challenge is to appropriately use and convey this uncertainty to policy makers and other data users, particularly to avoid underestimating the population impacted by hazards or other events.

## The Future of Bottom-Up Population Estimation

Bottom-up approaches for spatially disaggregated population estimation is a significant area of active research. In Nigeria, settlement mapping using very high-resolution imagery in combination with small-area microcensus surveys, geolocated national household surveys, and a range of geospatial layers, are being used to estimate population sizes and age and sex structures at a spatial resolution of 90 m (63). The outputs are being used to improve the efficiency and effectiveness of vaccination planning, forming the demographic basis of the Nigeria Vaccination Tracking System (vts.eocng.org/), as well as being adopted for humanitarian needs assessments (59). Elsewhere, the Afghanistan Central Statistics Organization, the United Nations Population Fund (UNFPA), WorldPop, and the Flowminder Foundation are using bottom-up approaches to derive population estimates across all parts of Afghanistan, as an update to existing estimates that are based on projections using the 1979 census and 2003–2005 household listing (64). The use of spatial statistics to quantify the relationships between microcensus-derived population counts and a range of spatial covariates, including detailed settlement information, is enabling population prediction in areas where recent enumeration has not been possible due to insecurity. Census enumeration generally fulfils a far broader role than the bottom-up population estimation approach we describe (census questionnaires also cover a range of more detailed demographic and socioeconomic factors), making direct-cost comparisons difficult. However, the approach we outline provides a low-cost option for the provision of national and subnational population estimates. For countries in Africa, a full-enumeration census typically costs approximately $1–2 per person; for example, the Ethiopia 2007 census was estimated to have cost $74 million, equating to around $1 per person (60). Following the work in Nigeria and Afghanistan, the bottom-up approach to estimating population counts, including acquiring microcensus and satellite imagery for the country, is estimated to cost between $0.03 and $0.15 per person in the population. There is also potential to broaden the bottom-up approach to cover further demographic and socioeconomic variables, with examples based on geolocated household survey data demonstrated elsewhere (65, 66), but more research in various settings is needed.

Subnational data on population remain central to government operations, and are vital for tracking progress toward national and international development goals. National population and housing censuses will continue to provide the most important source of such data, but in many cases these data are outdated and unreliable, with few other data sources, such as registries, available to aid in updates. Statistical approaches have been applied for the estimation of populations within specific urban and rural settings in the absence of census data (11, 33, 34, 61), and within refugee/internally displaced persons camps (39). However, there have so far been few attempts to provide estimates across national extents (5, 44) and less attention paid to low-income settings where contemporary population information is currently lacking. We propose that suitable data sources, computational power, and statistical methods are now available to enable high-resolution, spatially disaggregated national population estimation to be carried out in countries where comprehensive, recent, and reliable census data are unavailable and are unlikely to become available due to challenging contexts.
